# Individual Magnetoencephalography Response Profiles to Short-Duration L-Dopa in Parkinson’s Disease

**DOI:** 10.3389/fnhum.2021.640591

**Published:** 2021-03-15

**Authors:** Edgar Peña, Tareq M. Mohammad, Fedaa Almohammed, Tahani AlOtaibi, Shahpar Nahrir, Sheraz Khan, Vahe Poghosyan, Matthew D. Johnson, Jawad A. Bajwa

**Affiliations:** ^1^Department of Biomedical Engineering, University of Minnesota, Minneapolis, MN, United States; ^2^National Neuroscience Nursing Administration, King Fahad Medical City, Riyadh, Saudi Arabia; ^3^Department of Neurophysiology, National Neuroscience Institute, King Fahad Medical City, Riyadh, Saudi Arabia; ^4^Department of Neurology, National Neuroscience Institute, King Fahad Medical City, Riyadh, Saudi Arabia; ^5^Department of Radiology, Massachusetts General Hospital, Harvard Medical School, Boston, MA, United States; ^6^Athinoula A. Martinos Center for Biomedical Imaging, Massachusetts General Hospital, Harvard Medical School, Massachusetts Institute of Technology, Boston, MA, United States; ^7^McGovern Institute for Brain Research, Massachusetts Institute of Technology (MIT), Cambridge, MA, United States

**Keywords:** short duration L-Dopa response, magnetoencephalography, Parkinson’s disease, motor cortex, machine learning

## Abstract

Clinical responses to dopamine replacement therapy for individuals with Parkinson’s disease (PD) are often difficult to predict. We characterized changes in MDS-UPDRS motor factor scores resulting from a short-duration L-Dopa response (SDR), and investigated how the inter-subject clinical differences could be predicted from motor cortical magnetoencephalography (MEG). MDS-UPDRS motor factor scores and resting-state MEG recordings were collected during SDR from twenty individuals with a PD diagnosis. We used a novel subject-specific strategy based on linear support vector machines to quantify motor cortical oscillatory frequency profiles that best predicted medication state. Motor cortical profiles differed substantially across individuals and showed consistency across multiple data folds. There was a linear relationship between classification accuracy and SDR of lower limb bradykinesia, although this relationship did not persist after multiple comparison correction, suggesting that combinations of spectral power features alone are insufficient to predict clinical state. Factor score analysis of therapeutic response and novel subject-specific machine learning approaches based on subject-specific neuroimaging provide tools to predict outcomes of therapies for PD.

## Introduction

Parkinson’s Disease (PD) symptoms differ across individuals (Foltynie et al., 2002), exhibiting distinct symptom profiles that include motor and non-motor components (Goetz et al., 2008). Since therapeutic response may depend on the individual symptom profile or disease subtype (Marras et al., 2020), an understanding of how symptom profiles respond to therapies is needed to facilitate personalized therapeutic targeting. However, there is limited quantitative information on symptom profile response to treatment, including for the gold standard therapy L-Dopa where differential effects on motor signs are common to clinical observation (Sethi, 2008). While previous studies investigated the effects of short-duration L-Dopa response (SDR) across a subset of motor signs (Koller et al., 1989; Gomez Arevalo et al., 1997; Bartels et al., 2003), these studies did not provide quantitative information on SDR across the range of motor factors that are understood to characterize PD (Goetz et al., 2008).

Non-invasive neuroimaging can complement standard clinical outcome measures in managing neurodegenerative disorders by providing objective neurophysiological features to evaluate treatment efficacy that can be monitored over the course of therapy (Ross and Tabrizi, 2011; Weingarten et al., 2015; ten Kate et al., 2018). However, similar to clinical symptoms, non-invasive neuroimaging metrics also differ across individuals (Finn et al., 2015; Yang et al., 2017) and the therapeutic implications of such inter-subject variability in PD are currently not well characterized (Marras et al., 2020). Previous studies using non-invasive magnetoencephalography (MEG) in PD to image motor cortex reported SDR effects in a variety of neuroimaging metrics such as spectral power (Heinrichs-Graham et al., 2014; Cao et al., 2020; Vinding et al., 2020), cortico-muscular coherence (Salenius et al., 2002; Hirschmann et al., 2013), and interhemispheric coherence (Heinrichs-Graham et al., 2014). However, conflicting findings reflect a lack of robustness of any single metric when averaged across multiple subjects (Boon et al., 2019; Vinding et al., 2020), and the role of inter-subject variability remains unclear. Novel features may help account for outcome differences across individuals, as highlighted in recent MEG studies of healthy subjects using novel whole-brain modeling and feature extraction techniques (Vidaurre et al., 2018; Becker et al., 2020; Hirschmann et al., 2020). Nevertheless, inter-subject variability among multi-faceted neuroimaging metrics remains a challenge for identification of SDR biomarkers.

One potential strategy to leverage inter-subject variability among multiple simultaneous metrics is to adapt concepts from multivariate decoding previously used in other neuroimaging applications (Haynes and Rees, 2006, p. 200; Norman et al., 2006). Multivariate decoding trains classifiers to distinguish the effects of a given stimulus using multiple selected metrics, or features, simultaneously. Within a given study, the resulting classification accuracy can then represent the extent to which candidate features encode information related to the stimulus (Haynes, 2015; Hebart and Baker, 2018). In the context of SDR, the stimulus is L-Dopa, and the classification features can be any candidate set of metrics of clinical interest (e.g., spectral power in distinct frequency bands). Accuracies from subject-specific classification may then represent the degree to which the metrics chosen encode neurophysiological SDR effects across subjects. Finally, by quantifying the relationship between accuracies and clinical outcomes, one can evaluate the potential of a candidate feature *set* as a biomarker of clinical improvement.

Our study had three primary objectives: (1) characterize inter-subject variability of SDR in terms of motor profiles, (2) characterize inter-subject variability of SDR in terms of spectral power profiles across multiple frequency bands, (3) evaluate the ability of spectral power profiles to account for motor profile score changes in SDR. We quantified motor sign response profiles by applying the previously determined MDS-UPDRS motor factor structure (Goetz et al., 2008). We quantified MEG spectral profile changes using a novel subject-specific strategy that used power from multiple frequency bands within each subject to identify the combination of frequency bands that best classified whether a given subject was off or on L-Dopa. We interpreted classification accuracy as reflective of the degree to which frequency bands changed during SDR, and we interpreted frequency band weights as the relative importance of a given band in SDR. This method contrasts with the traditional strategy in MEG for PD of averaging data across all subjects (e.g., from single frequency bands). Finally, we calculated the relationship between motor factor SDR and classification accuracy to evaluate spectral power profiles as potential biomarkers of clinical SDR effects.

## Materials and Methods

The study protocol and consent form were approved by the King Fahad Medical City and University of Minnesota institutional review boards. The study followed ethical principles described in the Declaration of Helsinki. MDS-UPDRS Part III ratings were measured from 20 subjects with PD diagnosis per UK Brain Bank criteria (Gibb and Lees, 1988) (demographic information in Supplementary Table 1) after an overnight medication withdrawal (LEVODOPA-OFF) and again 1 h following administration of two carbidopa/L-Dopa tablets (25/100 mg) (LEVODOPA-ON). MDS-UPDRS factor scores (Goetz et al., 2008) were then calculated for (1) midline function, (2) rest tremor, (3) rigidity, (4) bradykinesia right upper extremity, (5) bradykinesia left upper extremity, (6) postural and kinetic tremors, (7) lower limb bradykinesia (see Supplementary Table 2). A history of dyskinesias was an exclusion criterion. During the MEG recordings, no motor fluctuations or dyskinesias were evident from video monitoring. We measured MMSE scores to confirm that no subjects were demented (Supplementary Table 1).

MEG, electrocardiogram (ECG), and electrooculogram (EOG) recordings were performed in LEVODOPA-OFF and LEVODOPA-ON conditions while each subject sat upright in an eyes-open resting-state. A 306-channel whole-head system (Vectorview; Elekta Neuromag Oy; Helsinki, Finland) within a magnetically shielded room (Ak3b Series; VAC; Hanau, Germany) streamed recordings at 1 kHz sampling rate (0.03–330 Hz bandpass analog filter). We digitized the head surface and fiducials for coregistration to MRI data. Subjects were asked to fixate on a particular point on the wall. We did not perform eye tracking. The head position was monitored continuously using head coils. External, internal, and movement-related noise was suppressed using temporally extended signal space separation (correlation limit: 0.98; data buffer length: 10 s) and movement correction algorithms in Maxfilter software (Elekta Neuromag Oy, Helsinki, Finland) (Taulu et al., 2004; Taulu and Simola, 2006). T1-weighted anatomical MRIs were acquired using an FSPGR sequence on 1.5-T GE scanners (GE Healthcare, Milwaukee, WI). All acquired MRIs were then processed using the automated reconstruction processing stream in FreeSurfer software (Dale et al., 1999; Fischl et al., 1999), which produces standard images with uniform and normalized intensities, and 1 mm isotropic resolution.

We inspected signals in Fieldtrip toolbox^1^ and rejected noisy channels and noisy 5 s epochs (ft_reject_visual), defining “noisy” as having an average 50–330 Hz power greater than 1.5 standard deviations above the mean. This frequency-domain approach targeted epochs contaminated with potential muscle artifacts and spike artifacts, and was used in place of a standard time-domain threshold for artifact detection. We subsequently imported signals into Brainstorm software (Tadel et al., 2011)^2^, along with the indices of rejected epochs and channels, and removed cardiac and blink artifacts using signal space projection with ECG and EOG events (Tesche et al., 1995). We coregistered the surface of the MRI with MEG sensor headpoints, and mapped MEG planar gradiometer signals onto a the cortical surface (downsampled to 15,000 vertices) using an overlapping spheres head model (Huang et al., 1999), no noise modeling, and whitened minimum norm estimate (Tadel et al., 2011) with sources constrained to be normal to the cortical surface. Other parameters had default Brainstorm values (signal-to-noise ratio: 3; noise covariance regularization: 0.1; depth weighting order: 0.5; maximal depth weighting: 10). We manually selected a region consisting of 40 vertices over the motor hand knob on each hemisphere in each subject (see Supplementary Table 3 for average MNI coordinate of vertex groups), and lowpass filtered the resulting data to remove high frequencies (Butterworth filter, order: 18, half-power frequency: 48 Hz). Source-space signals from each vertex in each region were split into 5 s long snippets with 50% overlap. After normalizing snippets to zero mean and unit variance, we calculated power spectra using Welch’s method (Hamming window length: 1 s; overlap: 50%; frequency resolution: 0.5 Hz). We averaged the power spectra from vertices within each vertex group, resulting in one power spectrum estimate per snippet per hemisphere in each subject. Source-space analyses excluded subjects 2, 5, and 9 due to strong artifacts from muscle, tremor, or corrupted MRI, respectively. Each subject had approximately 3 min of resting state data from each hemisphere and for each medication state.

Linear support vector machines (SVM) were constructed and trained on frequency band power to distinguish between LEVODOPA-OFF and LEVODOPA-ON within-subject. Subject-specific SVMs were trained and tested using power from 7–13, 13–20, 20–30, 35–45 Hz (Priori et al., 2004), avoiding high frequency artifacts and providing four bands that included frequencies previously investigated in PD neurophysiology (Priori et al., 2004; de Hemptinne et al., 2015). We standardized SVM feature inputs to the same scale (i.e., zero mean, unit variance) and constructed a linear SVM (fitcsvm on MATLAB R2018a; kernel scale: 1; standardize: false; kernel function: linear). To train the SVM, we separated the dataset into 10-fold and used 90% of the data for training and 10% of the data for testing for all possible combinations of folds. To ensure distinct training and testing datasets during cross-validation, we did not shuffle the data prior to assigning folds. We used one-dimensional Bayesian optimization to select the SVM box constraint (i.e., the soft margin SVM hyperparameter “C”), which was the only hyperparameter needed for linear SVM. We selected the box constraint value within the range of 1e−6 to 1e+6 that minimized 10-fold cross validation loss of the trained SVMs (bayesopt in MATLAB 2018a; acquisition function name: expected improvement plus; maximum objective evaluations: 30). Therefore, optimal box constraint values differed across subjects, but not across folds within subjects. We balanced the number of snippets for LEVODOPA-OFF and LEVODOPA-ON, such that there were equal numbers of snippets from each class available for training and testing. Linear regression between SVM accuracy and individual motor factor score changes quantified the degree to which neurophysiological profile changes reflected the clinical changes with medication state.

## Results

### Motor Factor Score Response to L-Dopa

**FIGURE 1:**
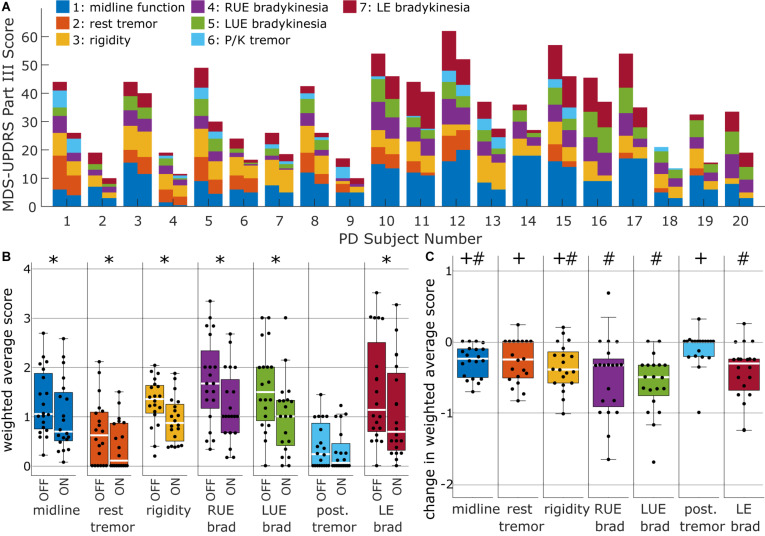
Clinical heterogeneity of responses to L-Dopa therapy across subjects. **(A)** MDS-UPDRS motor factor score profiles of short-duration L-Dopa responses across all subjects. Color-coding by motor factors represents the raw sum of item scores that belonged in a given motor factor according to (Goetz et al., 2008). Motor factors 1–7 consisted of 8, 6, 5, 3, 3, 4, and 4 MDS-UPDRS Part III items, respectively (see Supplementary Table 2). **(B)** Factor scores expressed as weighted averages of the individual items, where weightings were obtained from Goetz et al. (2008) (see Supplementary Table 2). Weighted average scores are shown in the same scale as individual MDS-UPDRS items (i.e., 0—Normal, 1—Slight, 2—Mild, 3—Moderate, 4—Severe). Asterisks denote factors in which SDR was significant in terms signed rank tests and Bonferroni correction for seven multiple comparisons. **(C)** Change in weighted average scores. Significant differences from *post hoc* Wilcoxon signed rank tests with Bejamin-Hochberg correction are denoted by either “#” (if different from postural tremor) or “+” (if different from left upper extremity bradykinesia).

Categorizing the MDS-UPDRS Part III scores into sub-items based on motor factors revealed a variety of motor profiles across subjects (Figure 1A). Right and left upper extremity bradykinesia (factors 4 and 5) had the highest weighted average factor scores at baseline (Figure 1B), although the median scores were only slightly smaller for midline, rigidity, and lower extremity bradykinesia (factors 1, 3, and 7). Tremor factors (factors 2 and 6) had the lowest weighted average factor scores. Short-duration levodopa significantly improved all factors except factor 6 (postural/kinetic tremor) (Wilcoxon signed rank test at α = 0.05; Bonferroni correction for seven comparisons) (Figure 1B). Nevertheless, responses were more pronounced for left upper extremity bradykinesia than for midline function, rest tremor, and rigidity (Figure 1C; Friedman *F*-test: *p* = 3.7e−4, df = 6; *post hoc* Wilcoxon signed rank test at α = 0.05; Benjamin-Hochberg correction for 21 multiple comparisons critical p: 0.014). We calculated a Spearman correlation matrix of the degree of SDR across factor scores, showing SDRs were uncorrelated across all motor factors (Supplementary Figure 1).

### Magnetoencephalographic Response to L-Dopa

**FIGURE 2:**
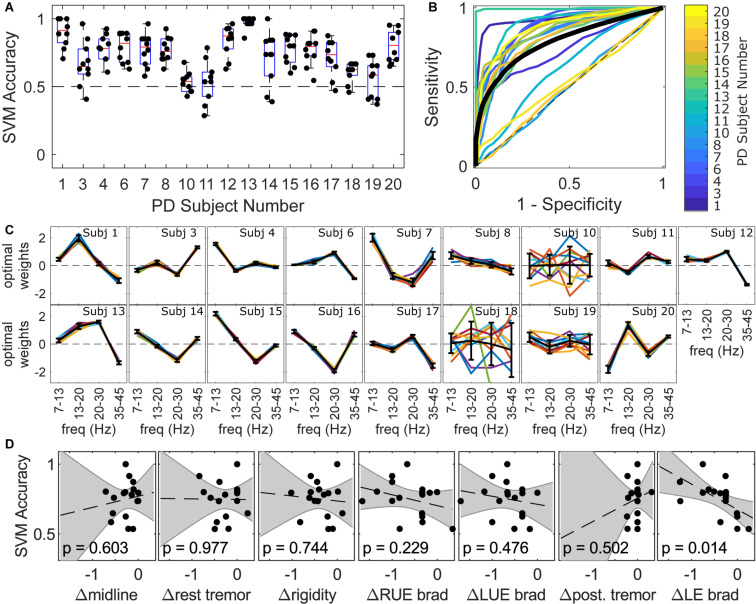
Individual profiles of MEG responses in terms of frequencies that best distinguished between LEVODOPA-OFF and LEVODOPA-ON within subjects. **(A)** Accuracy of within-subject linear support vector machine (SVM) in distinguishing between LEVODOPA-OFF and LEVODOPA-ON with 10-fold cross validation. Each dot is a separate fold. An accuracy of 0.5 is a random-chance prediction (black dashed line). **(B)** Average Receiver Operating Characteristic (ROC) curves across folds for each subject (colored lines) and the grand average ROC curve across subjects (black solid line). The dashed black diagonal line denotes the chance line. **(C)** MEG response profiles in terms of frequency band weights for each individual subject across all folds (colored lines) and averages across folds (bold black lines). Features were standardized to zero mean and unit variance. Positive weights reflect increase due to medication. Error bars denote the standard deviation of weights across folds. **(D)** Linear regression between median classification accuracy from **(A)** and clinical change in factor scores. Dashed lines show the linear fit for each factor. Shaded areas show the 95% confidence interval of the linear fit (*p*-values and confidence intervals not adjusted for multiple comparisons). Subjects 2, 5, and 9 were not included in SVM analysis due to prominent muscle, tremor, or imaging artifacts, respectively.

Within-subject linear SVMs distinguished LEVODOPA-OFF from LEVODOPA-ON at median classification accuracies ranging from 0.53 to 1.0 (Figure 2A), with fold classification accuracies being consistently above chance (>0.5) in most subjects (10/17). Within-subject receiver operating characteristic (ROC) curves were above the chance line in 14/17 subjects (Figure 2B). Linear SVMs identified the linear combination of bilateral motor cortical power from four frequency bands (i.e., [7–13 Hz], [13–20 Hz], [20–30 Hz], [35–45 Hz]) that best distinguished medication state, producing weights for each frequency band related to its importance in classification. MEG SDR response profiles, as represented by these frequency band weights, exhibited marked inter-subject diversity (Figure 2C) despite within-subject weights being relatively consistent during cross-validation tests in 13/17 subjects (Figure 2C). Feature weights were consistent across folds for most subjects, although certain subjects exhibited highly variable feature weights across folds (e.g., Subjects 10, 18, and 19). Without correction for multiple comparisons, there was a linear relationship between classification accuracy and SDR of lower limb bradykinesia across all 17 trained SVMs (*R*^2^ = 0.343; unadjusted *p* = 0.014; Figure 2D), although no motor factor correlated with classification accuracy after Bonferroni correction.

## Discussion

Despite the growing recognition of the need for personalized PD therapies (Espay and Lang, 2018; Marras et al., 2020), there is limited quantitative information on therapeutic response in the context of multi-faceted clinical and neurophysiological profiles. Our quantification of response profiles revealed substantial inter-subject variability in motor and magnetoencephalographic responses during SDR. Our results contribute quantitative characterizations of the multi-faceted PD response during SDR, further substantiating the established clinical observation that L-Dopa is most effective for bradykinesia and less often effective for postural tremor and midline motor signs including freezing of gait (Sethi, 2008). The results additionally motivate the use of factor scores to clarify the nature of responses to other therapies that can complement L-Dopa in personalized disease management. This is particularly important given that the symptoms associated with most severe impairment in advanced PD are those which do not respond well to L-Dopa (Hely et al., 2005). Notably, motor factor scores did not correlate with each other, and this independence amongst scores challenges the use of summated MDS-UPDRS III ratings as a primary clinical outcome measure, as motor factor scores may provide distinct clinical information about therapeutic response. While our PD cohort was of comparable size to previous MEG studies (Heinrichs-Graham et al., 2014; Cao et al., 2020; Vinding et al., 2020), future studies that leverage the factor score methodology in larger cohorts will be crucial to expand the needed quantitative information of therapeutic response in SDR, long-duration L-Dopa response (Anderson and Nutt, 2011; Cilia et al., 2020), and other interventional therapies.

Linear SVM identified the frequency bands that best distinguished LEVODOPA-OFF from LEVODOPA-ON, and frequency band weights revealed substantial inter-subject variability in MEG changes during SDR. Previous resting state MEG studies that investigated spectral power changes in motor cortex averaged changes across subjects and reported either an increase in spectral power [i.e., at either 14–30 Hz (Heinrichs-Graham et al., 2014) or 18–30 Hz (Cao et al., 2020)] or no change in spectral power (i.e., at 13–30 Hz; Vinding et al., 2020) during SDR. In contrast to the approach of averaging changes across subjects, we leveraged within-subject classifiers and the entire MEG time series to reveal within-subject response profiles. These within-subject response profiles suggested that subjects exhibited spectral changes due to SDR, and these changes had distinct frequency profiles rather than occurring at a single consistent frequency as assumed by traditional analysis methods. Indeed, while some subjects had response profiles that resembled those of other subjects (e.g., Figure 2C, Subjects 1 and 13 or Subjects 14 and 16), no single profile generalized across all subjects (Supplementary Figure 2). Notably, our use of subject-specific box constraints may prevent direct comparison of the magnitudes of feature weights across subjects, and preliminary comparisons of individual feature weights to motor factor scores showed no correlation.

In general, we did not identify strong relationships between classification accuracy and clinical motor scores, as only lower extremity bradykinesia correlated with classification accuracy prior to multiple comparison correction. This result is informative, suggesting that subject-specific changes in motor cortical frequency band power are insufficient to predict clinical changes due to SDR in heterogeneous cohorts. The findings motivate evaluation of alternative sets of candidate biomarkers, such as bursts of oscillatory activity (Vinding et al., 2020) and neural states derived from Hidden Markov Models of whole-brain signals (Hirschmann et al., 2020). The subject-specific classification method used here can be readily adapted to evaluate other sets of candidate features. Nevertheless, a number of caveats can complicate interpretation of classifier accuracy (Haynes, 2015; Hebart and Baker, 2018), and related multivariate methods such as representational similarity analysis (Kriegeskorte, 2008; Guggenmos et al., 2018) may provide complementary information to evaluate sets of candidate biomarkers in terms of direct profile differences between conditions.

An experimental limitation was the lack of counterbalance between the MEG data in LEVODOPA-OFF and LEVODOPA-ON conditions, which were always collected on the same visit in the same order. Thus, factors not related to the medication may have influenced classification (e.g., environment, subject familiarity with protocol). Addressing this important and challenging limitation may require alternative study designs involving multiple sessions per subject (e.g., training and testing on data recorded from different days). Furthermore, an inherent limitation of the present design is the potential difference in movement-related noise levels between LEVODOPA-OFF and LEVODOPA-ON conditions despite head movement compensation through continuous head localization. Additionally, the present analysis included both hemispheres to compare predictions against the largely bilateral set of clinical factor scores considered, thus neglecting potential relationships between the lateralized clinical factors of upper extremities and the laterality of MEG in PD (Heinrichs-Graham et al., 2017). Finally, while our recording duration (∼3 min) was consistent with prior resting-state spectral analyses (Pollok et al., 2012; Hall et al., 2014; Cao et al., 2020; Vinding et al., 2020), other relevant metrics, such as resting-state canonical brain networks, may require longer recording durations (Liuzzi et al., 2017).

We limited our classifications to linear SVMs and a small feature space that facilitated interpretability. Nevertheless, we recognize that other classification algorithms may be more appropriate to exploit specific relationships between features. While preliminary visual inspection of features did not reveal salient relationships to exploit, higher classification accuracy may be possible using non-linear kernel-based SVMs, or using alternative methods such as classification and regression trees. With more sophisticated methods, alternative techniques for interpretation are warranted (Haufe et al., 2014). Notably, while our hyperparameter space was unidimensional, the use of Bayesian optimization is readily applicable when the hyperparameter space is multidimensional. Despite the limitations noted, the present study contributes quantitative information regarding inter-subject variability of clinical and neurophysiological SDR profiles. The study further demonstrates the potential for classification approaches to evaluate candidate multi-faceted biomarkers relative to the *spectrum* of PD clinical phenotypes (Marras et al., 2020).

## Data Availability Statement

The motor factor scores for all subjects, support vector machine frequency weights, and cross-validated accuracies for all subjects were uploaded to the Data Repository for U of M (DRUM) and will be persistently accessible to the public at the following DOI: https://doi.org/10.13020/r6af-pj55. Further, the MATLAB code used to train and test the SVMs is available at the following GitHub repository: https://github.com/eurypt/MEG-UPDRS.

## Ethics Statement

The studies involving human participants were reviewed and approved by the King Fahad Medical City Institutional Review Board and University of Minnesota Institutional Review Board. The patients/participants provided their written informed consent to participate in this study.

## Author Contributions

EP analyzed the data and wrote the manuscript. MDJ and JAB conceived, designed, and oversaw execution of the research project and manuscript writing. TMM and SN collected the clinical data. FA and TA collected the magnetoencephalography data. SK and VP were involved in design and review of data analysis and in organizing and revising the manuscript. All authors contributed to the article and approved the submitted version.

## Conflict of Interest

The authors declare that the research was conducted in the absence of any commercial or financial relationships that could be construed as a potential conflict of interest.

## Footnotes

1 https://www.fieldtriptoolbox.org/

2 http://neuroimage.usc.edu/brainstorm
