# Supplementary material for: Individual Magnetoencephalography Response Profiles to Short-Duration L-Dopa in Parkinson’s Disease
Source: Front Hum Neurosci. 2021 Mar 15;15:640591. doi: 10.3389/fnhum.2021.640591 (PMC8005574; doi:10.3389/fnhum.2021.640591)
Supplement: Supplementary file 1 [file Data_Sheet_1.PDF]

# Supplementary Information

## Subject Demographic Information

Supplementary Table 1. Parkinson's disease subject demographic information. No subject exhibited motor fluctuations or dyskinesia during the MEG recordings. Subjects 1, 3, and 6 were illiterate and thus could not complete the full Mini-Mental Scores test.

| Subject | Sex | Age (years) | Disease Duration (years) | Levodopa Duration (years) | Hoehn-Yahr Stage | Mini-Mental Score |
|---|---|---|---|---|---|---|
| **1** | F | 70 | 5 | 4 | 2 | 20/25 |
| **2** | M | 58 | <1 | <1 | 2 | 26/30 |
| **3** | M | 71 | 5 | 4 | 2 | 19/21 |
| **4** | F | 57 | 2 | 1 | 2 | 30/30 |
| **5** | M | 67 | 7 | 6 | 2 | 29/30 |
| **6** | F | 62 | 9 | 8 | 2 | 28/28 |
| **7** | M | 55 | 6 | 5 | 2 | 29/30 |
| **8** | M | 71 | 6 | 6 | 2.5 | 30/30 |
| **9** | M | 76 | 5 | 3 | 1 | 30/30 |
| **10** | M | 55 | 5 | 5 | 2 | 30/30 |
| **11** | M | 70 | 7 | 7 | 3 | 30/30 |
| **12** | F | 55 | 9 | 7 | 3 | 27/30 |
| **13** | M | 51 | 3 | <1 | 2 | 30/30 |
| **14** | M | 57 | 1 | 1 | 3 | 29/30 |
| **15** | F | 53 | 5 | 4 | 2 | 28/30 |
| **16** | M | 69 | 11 | 11 | 2 | 26/30 |
| **17** | M | 70 | 4 | 2 | 3 | 25/30 |
| **18** | M | 69 | 1 | 1 | 2 | 30/30 |
| **19** | M | 69 | 6 | 6 | 2 | 30/30 |
| **20** | M | 52 | 6 | 6 | 2 | 30/30 |
| **Summary** | **F: 5<br>M: 7** | **63 ± 8** | **5 ± 3** | **4.4 ± 2.8** | **2.2 ± 0.5** | |

## Motor Factor Score Calculation

We calculated each motor factor score as the weighted average of the items and weights listed in Supplementary Table 2.

Supplementary Table 2. MDS-UPDRS items contributing to each factor score, and weights used in weighted averaging to obtain factor score. Weights were obtained from item loadings reported in [1].

| **MDS-UPDRS Item** | **Weight** | **Factor** |
|---|---|---|
| Speech | 0.59 | 1 |
| Facial Expression | 0.53 | 1 |
| Arising from Chair | 0.77 | 1 |
| Gait | 0.87 | 1 |
| Freezing of Gait | 0.83 | 1 |
| Postural Stability | 0.81 | 1 |
| Posture | 0.7 | 1 |
| Global Spontaneity of Movements | 0.64 | 1 |
| Rest Tremor Amplitude RUE | 0.72 | 2 |
| Rest Tremor Amplitude LUE | 0.71 | 2 |
| Rest Tremor Amplitude RLE | 0.73 | 2 |
| Rest Tremor Amplitude LLE | 0.71 | 2 |
| Rest Tremor Amplitude Lip Jaw | 0.59 | 2 |
| Rest Tremor Constancy | 0.88 | 2 |
| Rigidity Neck | 0.67 | 3 |
| Rigidity RUE | 0.73 | 3 |
| Rigidity LUE | 0.74 | 3 |
| Rigidity RLE | 0.8 | 3 |
| Rigidity LLE | 0.81 | 3 |
| Finger Tapping R | 0.67 | 4 |
| Hand Movements R | 0.66 | 4 |
| Pronation Supination Hands R | 0.68 | 4 |
| Finger Tapping L | 0.69 | 5 |
| Hand Movements L | 0.72 | 5 |
| Pronation Supination Hands L | 0.65 | 5 |
| Postural Tremor Hands R | 0.66 | 6 |
| Postural Tremor Hands L | 0.72 | 6 |
| Kinetic Tremor Hands R | 0.81 | 6 |
| Kinetic Tremor Hands L | 0.8 | 6 |
| Toe Tapping R | 0.65 | 7 |
| Toe Tapping L | 0.63 | 7 |
| Leg Agility R | 0.64 | 7 |
| Leg Agility L | 0.62 | 7 |

Supplementary Table 3. Average MNI coordinate of vertices selected on each hemisphere of each subject included in source-space analysis. MNI coordinates were based on an affine transformation of individual MRI to template in MNI space, as performed in Brainstorm software.

| Subject Number | Average MNI Coordinate of Vertices on Left Hemisphere (in mm) | | | Average MNI Coordinate of Vertices on Right Hemisphere (in mm) | | |
|---|---|---|---|---|---|---|
| 1 | -35 | -12 | 68 | 41 | -11 | 65 |
| 3 | -39 | -12 | 60 | 39 | -12 | 63 |
| 4 | -36 | -17 | 63 | 36 | -15 | 68 |
| 6 | -41 | -14 | 62 | 38 | -10 | 64 |
| 7 | -42 | -8 | 66 | 40 | -6 | 68 |
| 8 | -44 | -21 | 68 | 38 | -19 | 72 |
| 10 | -47 | -10 | 51 | 44 | -12 | 60 |
| 11 | -40 | -9 | 60 | 41 | -12 | 63 |
| 12 | -44 | -9 | 55 | 43 | -4 | 49 |
| 13 | -43 | -18 | 69 | 47 | -15 | 65 |
| 14 | -40 | -6 | 60 | 36 | -11 | 63 |
| 15 | -38 | -7 | 60 | 41 | -1 | 55 |
| 16 | -42 | -10 | 63 | 44 | -14 | 69 |
| 17 | -48 | -5 | 52 | 40 | -7 | 63 |
| 18 | -40 | -23 | 75 | 41 | -21 | 71 |
| 19 | -38 | -31 | 69 | 40 | -28 | 65 |
| 20 | -32 | -19 | 71 | 29 | -17 | 73 |

## Supplementary Information Figures

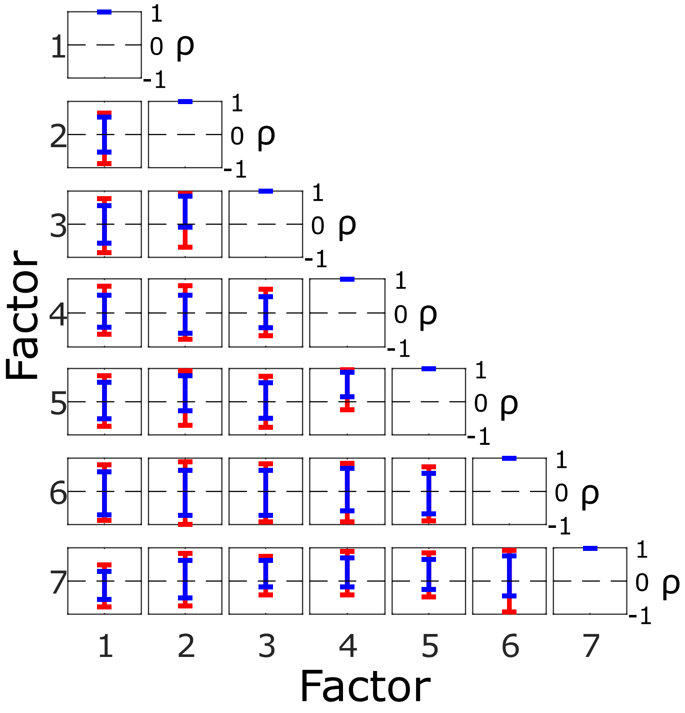

Supplementary Figure 1. Spearman correlation matrix across motor factor scores and 95% confidence intervals without (blue) and with (red) Bonferroni correction for 21 cross-factor comparisons. Confidence intervals of Spearman's rho were calculated using bootstrapping in MATLAB 2018a (bootci).

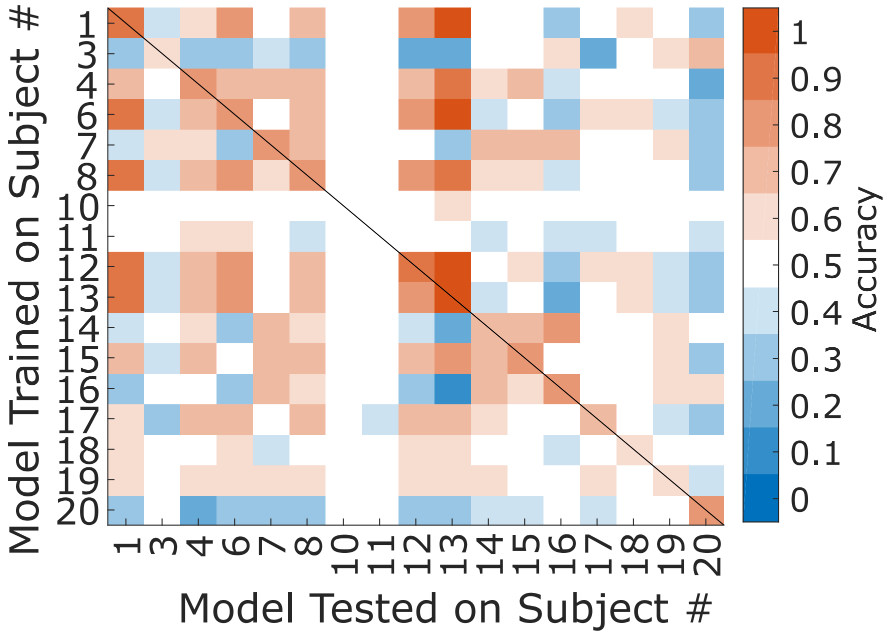


Supplementary Figure 2. Subject-specific classifier performance when tested on other subjects in the cohort on whom each classier was not trained. The diagonal elements along the black solid line show median 10-fold cross-validated accuracy within-subject classification.

## Supplementary Information References


[1] C. G. Goetz *et al.*, "Movement Disorder Society-sponsored revision of the Unified Parkinson's Disease Rating Scale (MDS-UPDRS): Scale presentation and clinimetric testing results," *Mov. Disord.*, vol. 23, no. 15, pp. 2129–2170, Nov. 2008, doi: 10.1002/mds.22340.

[2] C. D. Tesche, M. A. Uusitalo, R. J. Ilmoniemi, M. Huotilainen, M. Kajola, and O. Salonen, "Signal-space projections of MEG data characterize both distributed and well-localized neuronal sources," *Electroencephalogr. Clin. Neurophysiol.*, vol. 95, no. 3, pp. 189–200, Sep. 1995, doi: 10.1016/0013-4694(95)00064-6.

[3] A. M. Dale, B. Fischl, and M. I. Sereno, "Cortical Surface-Based Analysis," *NeuroImage*, vol. 9, no. 2, pp. 179–194, Feb. 1999, doi: 10.1006/nimg.1998.0395.

[4] B. Fischl, M. I. Sereno, and A. M. Dale, "Cortical Surface-Based Analysis," *NeuroImage*, vol. 9, no. 2, pp. 195–207, Feb. 1999, doi: 10.1006/nimg.1998.0396.

[5] M. X. Huang, J. C. Mosher, and R. M. Leahy, "A sensor-weighted overlapping-sphere head model and exhaustive head model comparison for MEG," *Phys. Med. Biol.*, vol. 44, no. 2, pp. 423–440, Feb. 1999.

[6] F. Tadel, S. Baillet, J. C. Mosher, D. Pantazis, and R. M. Leahy, “Brainstorm: A User-Friendly Application for MEG/EEG Analysis,” *Comput. Intell. Neurosci.*, vol. 2011, pp. 1–13, 2011, doi: 10.1155/2011/879716.
